# Slow-inactivated sodium channels as a therapeutic target in trigeminal neuralgia: evidence from a systematic review and meta-analysis of lacosamide

**DOI:** 10.22514/jofph.2026.037

**Published:** 2026-05-12

**Authors:** Tonporn Meechumnarn, Somkiat Phutinart, Prakit Anukoolwittaya, Waritnun Kleechaya, Patcharanan Deprasertwong, Patavee Pajareya, Abhishet Varama, Noppachai Siranart, Sekh Thanprasertsuk

**Affiliations:** ^1^Orofacial Pain Clinic, Department of Oral Medicine, Faculty of Dentistry, Chulalongkorn University, 10330 Bangkok, Thailand; ^2^Faculty of Medicine, Chulalongkorn University, 10330 Bangkok, Thailand; ^3^Comprehensive Headache and Orofacial Pain (CHOP) Service and Research Group, Chulalongkorn University, 10330 Bangkok, Thailand; ^4^Division of Neurology, Department of Medicine, Faculty of Medicine, Chulalongkorn University, 10330 Bangkok, Thailand; ^5^Department of Physiology, Faculty of Medicine, Chulalongkorn University, 10330 Bangkok, Thailand

**Keywords:** Trigeminal neuralgia, Antiseizure medication, Lacosamide

## Abstract

**Background**: Lacosamide (LCM), a selective enhancer of the slow inactivation of voltage-gated sodium channels, has been proposed as a treatment for trigeminal neuralgia (TN), although its clinical efficacy and safety remain incompletely defined. **Methods**: We conducted a systematic review and meta-analysis of cohort studies involving adults with TN. PubMed, EMBASE, and CENTRAL were searched from inception to 16 October 2025. The primary outcomes were efficacy, measured as pain relief, and safety, assessed by adverse events (AEs) associated with LCM. **Results**: Four studies were included in the quantitative synthesis. The pooled proportion of patients achieving pain relief with oral LCM was 66.7% (95% confidence interval (CI): 57.3%–74.9%; *I*^2^ = 0.0%). The pooled overall incidence of adverse events with oral LCM was 35.2% (95% CI: 26.8%–44.6%; *I*^2^ = 20.7%). The most frequently reported adverse event of oral LCM was sleepiness/somnolence (24.6%), followed by dizziness (21.7%), instability (2.5%), first-degree atrioventricular block (1.7%), and inattention (1.7%). All adverse events were infrequent and generally non-persistent. Additionally, intravenous LCM in acute exacerbation of TN reported that 77.8% of patients achieved patient-reported pain absence within 10 hours, with only 1.6% experiencing sleepiness. **Conclusion**: Lacosamide (LCM) has shown favorable outcomes in some patients with trigeminal neuralgia (TN) and may serve as an alternative therapy, particularly those refractory to or intolerant of first-line agents, and intravenous LCM may be useful for acute exacerbations. However, the current evidence is preliminary, observational, and insufficient to support comparative treatment decisions. Randomized controlled trials are needed to establish its efficacy and safety. **The PROSPERO Registration**: CRD420261295578.

## 1. Introduction

Trigeminal neuralgia (TN) is characterized by unilateral, paroxysmal, electric-shock-like attacks arising within one or more divisions of the trigeminal nerve [1]. Contemporary population data suggest that the burden of TN is substantial and may be increasing over time [2]. The prevailing pathophysiological model implicates neurovascular compression at the trigeminal nerve root entry zone by an adjacent vessel, most commonly an artery; however, up to 10% of patients have no demonstrable compression on imaging, underscoring etiologic heterogeneity and therapeutic complexity [3].

First-line management remains pharmacotherapy, with carbamazepine (CBZ) widely recommended [4, 5]. However, its efficacy is often limited by dose-limiting adverse effects (*e.g.*, somnolence, dizziness, ataxia, hepatotoxicity, hyponatremia, and rash) and clinically relevant drug-drug interactions, prompting consideration of alternatives when efficacy wanes or intolerance develops [4, 6]. For medically refractory cases, microvascular decompression (MVD) offers durable pain relief but is invasive and dependent on surgical fitness, with risks including cranial nerve injury, infection, and cerebrospinal fluid leak [7, 8].

Lacosamide (LCM), a third-generation antiseizure medication (ASM), enhances slow inactivation of voltage-gated sodium channels (Na_V_) and thereby modulates pathologic neuronal hyperexcitability [9, 10]. Beyond epilepsy, LCM has been used in chronic neuropathic pain and other conditions, including painful diabetic neuropathy, small-fiber neuropathy, migraine, and short-lasting unilateral neuralgiform headache attacks with conjunctival injection and tearing (SUNCT) and with cranial autonomic symptoms (SUNA) [11, 12, 13, 14, 15, 16]. Recent clinical reports and reviews have proposed LCM as a candidate therapy for TN [9, 17, 18, 19, 20, 21]. Given these mechanistic considerations, the tolerability profile observed in neuropathic pain populations, and emerging clinical signals in TN, a rigorous synthesis of the evidence is warranted.

Accordingly, this systematic review and meta-analysis evaluates the efficacy and safety of LCM for TN to clarify the current evidence base for its potential clinical use.

## 2. Methods

### 2.1 Protocol and registration

This review was conducted in accordance with Cochrane guidance for systematic reviews of interventions and is reported according to the Preferred Reporting Items for Systematic reviews and Meta-Analyses (PRISMA) 2020 statement (**Supplementary material 1**) [22]. The protocol was prospectively registered in International Prospective Register of Systematic Reviews (PROSPERO) (CRD420261295578).

### 2.2 Eligibility criteria

ⅰ. Population

Adults diagnosed with TN according to the International Classification of Headache Disorders, 3rd edition (ICHD-3) [23] or the International Classification of Orofacial Pain, 1st edition (ICOP-1) [1] constituted the population for this study.

ⅱ. Intervention and Comparators

Lacosamide (any dose, route, or regimen) administered as monotherapy or adjunctive therapy was included. Eligible comparators, when present, included active pharmacologic agents (*e.g.*, carbamazepine (CBZ), oxcarbazepine (OXC), gabapentin (GBP), baclofen (BAC), and phenytoin (PHT)) or usual care.

ⅲ. Outcomes

At least one of the following outcomes was required: (1) pain outcomes (*e.g.*, at least 50% pain relief, change in pain intensity on the visual analog scale (VAS) or numerical rating scale (NRS), or Brief Pain Inventory-Facial (BPI-F)) or (2) safety outcomes (any adverse events, serious adverse events, or withdrawals due to adverse events).

ⅳ. Study Design

We included only prospective or retrospective cohort studies and excluded cross-sectional studies, case series, case reports, animal, and *in vitro* studies.

### 2.3 Information sources and search strategy

We searched PubMed, EMBASE, and the Cochrane Central Register of Controlled Trials from database inception to 16 October 2025. The strategy combined controlled vocabulary and free-text terms for the key concepts (“trigeminal neuralgia”, “lacosamide”, and “neuropathic pain”), applying field tags and adjacency/proximity operators as appropriate for each database. No language or publication-status restrictions were applied. To minimize retrieval bias, we also performed forward and backward citation tracking (via Scopus) of all included studies and relevant reviews. Full search strategies are provided in **Supplementary Table 1**.

### 2.4 Study selection

Two reviewers (PD, WK) independently screened titles and abstracts, followed by full-text assessment against the eligibility criteria. Discrepancies were resolved by discussion; when consensus was not reached, a third reviewer (NS) adjudicated. Reasons for exclusion at full-text screening were recorded, and the selection process is summarized in a PRISMA flow diagram.

### 2.5 Data extraction

Using a pre-piloted extraction form, PD and WK independently extracted: study identifiers; design and setting; sample size; eligibility criteria; participant characteristics (age, sex, etiology of secondary TN, and time since diagnosis); intervention details (dose, schedule, co-therapies); comparators; outcome definitions and measurement time points; effect estimates with precision; and funding and conflicts of interest. Disagreements were resolved by consensus; a third reviewer (SP) performed random verification for accuracy. Corresponding authors were contacted when critical data were missing or unclear. When studies reported medians and interquartile ranges, we estimated means and standard deviations using established methods, where appropriate. For multi-arm studies, shared comparators were handled according to Cochrane guidance to avoid double-counting.

### 2.6 Risk of bias assessment

Two reviewers independently assessed cohort studies using the Newcastle-Ottawa Scale and non-randomized controlled trials using the Cochrane Risk of Bias in Non-randomized Studies of Interventions, Version 2 (ROBINS-I V2) tool. The assessment results are presented in **Supplementary Table 2** (Ref. [9, 19, 20, 21]) and **Supplementary Fig. 1** (Ref. [9, 19, 20, 21]).

### 2.7 Statistical analysis

All analyses were conducted in R, version 4.3.1. To evaluate efficacy and safety, we performed single-arm meta-analyses of proportions (pooled responder rates and pooled adverse-event incidences). A common-effects model was used when between-study heterogeneity was not statistically significant, as determined by Cochran’s *Q* test (*p* > 0.05); otherwise, a random-effects model was applied. Results are presented as pooled proportions with 95% confidence intervals (CIs). Statistical heterogeneity was quantified using *I*^2^ and assessed with Cochran’s *Q* test. When pooling was not appropriate, findings were summarized narratively.

When potential population overlap was suspected among studies conducted in similar time periods and geographic regions, sensitivity analyses were performed, retaining only studies judged to include distinct populations.

### 2.8 Assessment of publication bias

Small-study effects were evaluated using visual inspection of funnel plots and Egger’s regression test.

## 3. Results

### 3.1 Study selection

From 937 records identified across databases, 608 remained after duplicates removal. Following title and abstract screening, 90 full-text articles were assessed for eligibility, and 4 studies met the inclusion criteria, contributing a total of 284 participants to the meta-analysis (PRISMA flow diagram in Fig. 1). Characteristics of the included studies are presented in **Supplementary Table 2**.

**Fig. 1.** PRISMA 2020 flow diagram.

### 3.2 Efficacy outcomes

In this systematic review, four studies evaluated the efficacy of LCM [9, 19, 20, 21]. All four studies were included in the qualitative synthesis. However, only two studies (*k* = 2) reported pain relief outcomes that were sufficiently comparable to be included in the pooled meta-analysis [9, 20]. The pooled proportion of patients treated with oral LCM who reported pain improvement without requiring additional treatment within 3 months was 66.7% (95% CI: 57.3%–74.9%; *I*^2^ = 0.0%) (Table 1; **Supplementary Fig. 2**, Ref. [9, 20]). The remaining studies reported efficacy using different outcome measures. One study assessed efficacy as a reduction in the BPI-F pain score and reported a 70% reduction over a 4-week period with oral LCM at a dose of 400 mg/day [19].

**Table 1.** Efficacy and safety of lacosamide in patients with trigeminal neuralgia.

Outcome	Outcome Subgroup	*k*	*I* ^2^	Proportion (95% CI)
Pain Relief	All	2	0.0%	66.7% (57.3%–74.9%)
Adverse Events				
	All	2	20.7%	35.2% (26.8%–44.6%)
	Sleepiness/Somnolence	3	91.7%	24.6% (0.4%–96.1%)
	Dizziness	3	35.5%	21.7% (9.6%–41.8%)
	Instability	3	0.0%	2.5% (0.8%–7.5%)
	First-degree AVB	3	0.0%	1.7% (0.4%–6.4%)
	Inattention	3	0.0%	1.7% (0.4%–6.4%)
	Bradycardia	3	0.0%	0.8% (0.1%–5.7%)
	Cutaneous Rash	3	0.0%	0.8% (0.1%–5.7%)
	Diplopia	3	0.0%	0.8% (0.1%–5.7%)
	Insomnia	3	0.0%	0.8% (0.1%–5.7%)
	Itchiness	3	0.0%	0.8% (0.1%–5.7%)
	Nausea	3	0.0%	0.8% (0.1%–5.7%)
	Tremor	3	0.0%	0.8% (0.1%–5.7%)

*k, number of included studies; AVB, atrioventricular block; CI, confidence interval.*

Another study evaluated intravenous (IV) LCM in an acute setting and reported that 77.8% of patients achieved patient-reported pain absence within 10 hours without requiring additional treatment [21].

### 3.3 Safety outcomes

In this systematic review, four studies reported adverse events (AEs) associated with LCM [9, 19, 20, 21]. All four studies were also included in the qualitative synthesis of safety outcomes. However, only two studies (*k* = 2) reported the overall incidence of AEs with oral LCM and were therefore included in the pooled analysis of overall AE incidence [9, 20]. The pooled overall incidence of AEs was 35.2% (95% CI: 26.8%–44.6%; *I*^2^ = 20.7%) (**Supplementary Fig. 3**, Ref. [9, 19, 20]).

In contrast, three studies (*k* = 3) reported individual AEs associated with oral LCM, allowing for pooled analyses of specific AE outcomes [9, 19, 20]. The most common AE was sleepiness/somnolence (24.6%), followed by dizziness (21.7%), instability (2.5%), first-degree atrioventricular block (1.7%), and inattention (1.7%). Other reported AEs, including cutaneous rash, diplopia, insomnia, itchiness, nausea, and bradycardia, each occurred in approximately 0.8% of patients (Table 1, **Supplementary Fig. 3**). Since two of the studies used for pooling the incidence of AEs potentially had overlapping populations [9, 20], sensitivity analyses were performed by excluding either study. Most findings were similar to the main analysis, with sleepiness/somnolence being the most common AE (26.7% and 46.8%) followed by dizziness (21.4% and 29.4%). The incidence of AEs other than somnolence/sleepiness and dizziness was low across analyses (**Supplementary Table 3**, Ref. [9, 19, 20]).

Among the remaining studies that used oral LCM with longer follow-up, the overall adverse event proportion ranged between 32.6% [20] and 45.5% [9]. Sleepiness/somnolence was reported in all oral LCM studies, with proportions of 4.7% [20], 22.7% [9], and 75.0% [19]. Dizziness was likewise reported across all oral LCM studies, ranging from 18.6% to 41.7% [9, 19, 20]. Instability and inattention were reported in only one study each, at 3.5% and 2.3%, respectively [20]. First-degree atrioventricular block was reported in two studies, with incidences of 1.2% [20] and 4.5% [9]. The remaining AEs occurred less frequently and were reported in only one of the included studies [20].

In the study evaluating IV LCM in the acute setting, AEs were rare, with only 1.6% of the cohort experiencing sleepiness [21]. Therefore, not all included studies contributed to each pooled outcome.

### 3.4 Publication bias

Although funnel plots and Egger’s regression test were examined, the small number of studies available for pooled analyses (*k* = 3) limits the reliability of formal publication bias assessment. Statistical tests for small-study effects are underpowered when fewer than ten studies are available; therefore, the possibility of publication bias cannot be excluded.

## 4. Discussion

To our knowledge, this systematic review and meta-analysis is the first to synthesize the clinical efficacy and safety of LCM in TN. Across the included studies, 66.7% of patients receiving oral LCM achieved clinically meaningful pain relief, while AEs associated with oral LCM occurred in 35.2% of patients overall and were generally mild and non-persistent. Somnolence/sleepiness and dizziness were the most frequently reported AEs with oral LCM, while other events occurred infrequently. Nevertheless, there was considerable heterogeneity in reported adverse event rates across studies, particularly for somnolence (ranging from 4.7% to 75%). This variation likely reflects differences in titration protocols and target doses, as more rapid escalation may increase dose-dependent central nervous system effects. In addition, baseline patient characteristics differed across studies; populations refractory to or intolerant of other sodium channel blockers may exhibit differing susceptibility to sedative effects compared with treatment-naïve patients. Differences in adverse event ascertainment methods and terminology (*e.g.*, “somnolence” *vs.* “sleepiness” *vs.* “drowsiness”) may further contribute to the observed variability. In addition, one study evaluating an IV formulation of LCM in an acute setting reported that 77.8% of patients reported pain absence within 10 hours, with only 1.6% experiencing sleepiness. Overall, these findings suggest that LCM may be a potential alternative or adjunctive treatment option for TN in both oral and IV formulations, due to its favorable balance between analgesic efficacy and tolerability.

The therapeutic signal is biologically coherent given current models of TN pathophysiology. Classical TN is typically driven by neurovascular compression at the trigeminal root entry zone, producing focal demyelination, axonal distortion, and ephaptic crosstalk that amplifies ectopic discharges [24, 25, 26]. Dysregulation of Na_V_ channels supports a channelopathy framework for pain generation [3, 25, 27].

LCM selectively enhances slow inactivation of Na_V_ channels without affecting fast inactivation [28, 29, 30], distinguishing it from CBZ, OXC, and PHT, which primarily promote fast inactivation [29]. Slow inactivation represents a more slowly developing non-conducting channel state that accumulates during sustained or repetitive depolarization [31, 32]. By promoting entry into this state and shifting its voltage dependence in the hyperpolarizing direction, LCM reduces the pool of available channels during periods of high-frequency firing, thereby suppressing pathological repetitive discharges while relatively sparing normal physiological conduction [32, 33]. This mechanism may confer a theoretical advantage in neuropathic conditions characterized by ectopic or high-frequency firing, such as TN, and plausibly underlies the clinical effects observed in the included studies [34].

Beyond TN, LCM has shown potential benefit across neuropathic pain conditions, with improvements reported in pain intensity and patient-reported outcomes, though effect sizes vary across populations [14, 35, 36]. In TN specifically, LCM’s mechanistic selectivity and tolerability profile make it particularly relevant in patients who are intolerant of, contraindicated for, or refractory to aromatic ASMs.

ASMs commonly used in TN (*e.g.*, CBZ, OXC, and PHT) carry a recognized risk of hypersensitivity reactions, an issue that is particularly salient in patients with a prior history of drug allergy or rash and in those with cross-reactivity among aromatic ASMs [37]. Current clinical guidance recommends genetic testing to identify alleles associated with severe cutaneous adverse reactions (SCARs), notably human leukocyte antigen allele A31:01 (HLA-A*31:01) in European and Japanese populations and HLA-B*15:02 in Asian populations, where CBZ/OXC-induced Stevens–Johnson syndrome/toxic epidermal necrolysis (SJS/TEN) and drug reaction with eosinophilia and systemic symptoms (DRESS) risk is increased [37, 38]. In such patients, non-aromatic alternatives are preferred; LCM, being non-aromatic, is therefore a rational substitute [37]. Although isolated cutaneous reactions to LCM have been reported, the incidence is low (approximately 0.36% and <1% in available series) [39, 40], and there is no established association with HLA-B*15:02. Early clinical experience, including a pilot study in HLA-B*15:02-positive patients, did not identify severe cutaneous reactions with LCM [19]. Collectively, these data support LCM as a potentially safer alternative in genetically at-risk or rash-prone patients, while still requiring routine dermatologic vigilance.

Taken together, the available evidence suggests that LCM may play a potential role in the treatment of TN, either as an alternative when first-line aromatic agents are not tolerated or are contraindicated, or as an adjunct when only a partial response persists. The overall AE incidence of 35.2%, dominated by transient symptoms (notably somnolence/sleepiness), suggests that careful titration and monitoring may optimize adherence while maintaining analgesic benefit.

### 4.1 Limitations

This review has several limitations. First, it is limited by the modest number of eligible studies and a relatively small, pooled sample size. In addition, only one study evaluated intravenous lacosamide; therefore, evidence regarding IV LCM remains highly preliminary and should be considered exploratory. Second, three of the included studies, two of which were part of the meta-analysis, originated from the same research group and geographic region, raising the possibility of overlapping patient cohorts. In particular, Muñoz-Vendrell *et al*. [20] (2023) and Muñoz-Vendrell *et al*. [9] (2025) may have overlapping inclusion criteria and enrollment periods, which could result in partial duplication of data and may influence the pooled estimates. Although study periods and clinical contexts differed, the potential for partial sample overlap cannot be entirely excluded, as the authors did not specify whether patients were recruited from the same cohort. Nevertheless, this potential overlap is likely to have only a modest effect on our findings, given that our study primarily aims to provide preliminary evidence supporting the adjunctive use of LCM in TN treatment. Third, Heterogeneity across AE estimates may reflect differences in prior exposure (primary use *vs.* refractory to CBZ), dosing strategies, and outcome ascertainment. Although some included studies reported active comparators (*e.g.*, CBZ, PHT, GBP, and BAC) with extractable effect measures, the comparator interventions were highly heterogeneous, and no intervention other than LCM was evaluated consistently across studies. For this reason, we performed only single-arm meta-analyses, which limited our ability to assess relative efficacy compared with conventional treatments. In addition, our meta-analysis did not perform comparisons with first-line therapies, such as CBZ, as most included studies involved patients who were intolerant of or refractory to first-line treatment. Therefore, direct comparisons with first-line therapies were not feasible. Finally, the observational design of the included studies limits causal inference, and estimates for specific AEs remain heterogeneous and imprecise. These constraints underscore the need for higher-quality evidence.

### 4.2 Future directions

Well-designed randomized controlled trials comparing LCM directly with conventional therapies (*e.g.*, CBZ) are needed, with standardized pain endpoints, predefined time points, and systematic AE monitoring (including adherence and discontinuation). Larger real-world studies should characterize efficacy across TN phenotypes, dosing regimens, and genetic risk strata, clarifying LCM’s role as an alternative or adjunct in TN. Additional high-quality evidence will be important in determining whether LCM should be considered in future clinical practice recommendations for TN.

### 4.3 Certainty of evidence

Using the GRADE framework (**Supplementary Table 4**), the overall certainty of evidence was rated as very low. The evidence was downgraded because of the observational study design, risk of bias, imprecision related to small sample size and wide confidence intervals, and potential publication bias. Therefore, confidence in the pooled estimates is limited, and future well-designed randomized controlled trials are highly likely to influence these findings.

## 5. Conclusion

In observational cohorts, oral LCM has been associated with favorable pain outcomes in some patients with TN and may serve as an alternative therapy, particularly for those who are intolerant of or refractory to first-line agents. Intravenous LCM may also offer a potential option for managing acute exacerbations in emergency settings. However, these findings are based on preliminary observational evidence and should not be interpreted as sufficient to support comparative treatment decisions or routine preference over established therapies. Given the limited number of non-randomized studies, risk of bias, small sample size, and imprecision in safety estimates, the overall certainty of evidence remains very low. Well-designed randomized controlled trials with standardized outcome measures are needed to determine the comparative efficacy, safety, and appropriate clinical role of LCM in TN.

## Supplementary Material

Supplementary material associated with this article can be found, in the online version, at https://files.jofph.com/ files/article/2054077232127590400/attachment/ Supplementary%20material.zip.




