# Supplementary material for: Slow-inactivated sodium channels as a therapeutic target in trigeminal neuralgia: evidence from a systematic review and meta-analysis of lacosamide
Source: J Oral Facial Pain Headache. 2026 May 12;40(3):76–82. doi: 10.22514/jofph.2026.037 (PMC13223906; doi:10.22514/jofph.2026.037)
Supplement: Supplementary file 2 [file Supplementary-material-2.docx]

Supplementary material 2

Supplementary Table 1. Search terms used in each database included in the meta-analysis.

| Database | Search Terms | Hits |
| --- | --- | --- |
| MEDLINE  (via PubMed) | ((“Lacosamide”[Mesh] OR lacosamide[tiab] OR “lacosamide sodium”[tiab] OR anticonvulsant*[tiab] OR antiepileptic*[tiab] OR “antiepileptic drug”[tiab]) AND (“Trigeminal Neuralgia”[Mesh] OR “trigeminal neuralgia”[tiab] OR “tic douloureux”[tiab] OR “facial neuropathic pain”[tiab] OR “trigeminal neuropathic pain”[tiab] OR “facial pain”[tiab] OR “trigeminal pain”[tiab] OR “neuropath* facial pain”[tiab] OR “cranial neuralgia”[tiab] OR “trigeminal nerve disorder”[tiab] OR “orofacial pain”[tiab] OR “nerve pain”[tiab])) | 382 |
| EMBASE  (via Scopus) | ((INDEXTERMS(Lacosamide) OR TITLE-ABS(lacosamide) OR TITLE-ABS(“lacosamide sodium”) OR TITLE-ABS(anticonvulsant*) OR TITLE-ABS(antiepileptic*) OR TITLE-ABS(“antiepileptic drug”)) AND (INDEXTERMS(“Trigeminal Neuralgia”) OR TITLE-ABS(“trigeminal neuralgia”) OR TITLE-ABS(“tic douloureux”) OR TITLE-ABS(“facial neuropathic pain”) OR TITLE-ABS(“trigeminal neuropathic pain”) OR TITLE-ABS(“facial pain”) OR TITLE-ABS(“trigeminal pain”) OR TITLE-ABS(“neuropath* facial pain”) OR TITLE-ABS(“cranial neuralgia”) OR TITLE-ABS(“trigeminal nerve disorder”) OR TITLE-ABS(“orofacial pain”) OR TITLE-ABS(“nerve pain”))) | 480 |
| Cochrane Database of Systematic Reviews | ([mh “Lacosamide”] OR lacosamide OR “lacosamide sodium” OR anticonvulsant* OR antiepileptic* OR “antiepileptic drug”) AND ([mh “Trigeminal Neuralgia”] OR “trigeminal neuralgia” OR “tic douloureux” OR “facial neuropathic pain” OR “trigeminal neuropathic pain” OR “facial pain” OR “trigeminal pain” OR (neuropath* NEAR/1 “facial pain”) OR “cranial neuralgia” OR “trigeminal nerve disorder” OR “orofacial pain” OR “nerve pain”) | 75 |

The search was carried out from the inception to October 2025.

Total search results from all databases: 937.

Mesh, Medical Subject Heading.

Supplementary Table 2. Baseline characteristics of studies included in the meta-analysis.

| Author, Year | Country/Center | Study Design | Population Definition | Setting | N | Age, yr | Female, % | Lacosamide Dose, mg, Route | Comparison, Route | Secondary Etiology, % | Time since TN diagnosis, Median (IQR) | Quality |
| --- | --- | --- | --- | --- | --- | --- | --- | --- | --- | --- | --- | --- |
| Munoz-Vendrell *et al*. [21], 2022 | Spain/emergency department of Bellvitge University Hospital, Barcelona, Spain | Retrospective cohort | Met diagnostic criteria for TN according to ICHD-3 | Patients with acute exacerbations of trigeminal neuralgia between 2012 and 2020 who presented to the emergency room received intravenous LCM or PHT. | 121 | Median: 61.0 (IQR: 65.0) | 66.1 | Median: 180 (Range: 50–400), IV | PHT, IV | 10.4 | 2.2 (6.4) | S3C2O3 |
| Munoz-Vendrell *et al*. [20], 2023 | Spain/Bellvitge University Hospital, Barcelona, Spain | Retrospective cohort | Met diagnostic criteria for TN according to ICHD-3 | Patients with trigeminal neuralgia between 2015 and 2021 were prescribed oral lacosamide for the symptomatic treatment of TN pain at any time during follow-up due to intolerance or refractoriness to first-line therapy. | 86 | Mean: 62.0 (SD: 15.6) | 63.0 | Median: 200 (IQR: 100), oral | N/A | 19.0 | 4.6 (7) | S2C0O3 |
| Lappichet-paiboon *et al*. [19], 2024 | Thailand/Orofacial Pain and Oral Medicine Clinic, Faculty of Dentistry, Khon Kaen University | Prospective controlled pilot clinical trial | Met diagnostic criteria for TN according to ICHD-3 | Patients with trigeminal neuralgia between April and December 2021 who had a pain score of 4 or higher on the numeric rating scale, and who had not previously received any treatment for TN or experienced pain after a natural remission period. | 18 | Mean: 60.9 (SD: 5.6) | 66.7 | 200 (n = 5) 400 (n = 7), oral | CBZ, Oral | N/A | N/A | S3C2O3 |
| Munoz-Vendrell *et al*. [9], 2025 | Spain/neurology clinic, Bellvitge University Hospital, Barcelona, Spain | Retrospective cohort | Met diagnostic criteria for TN according to ICHD-3 | Patients with trigeminal neuralgia between January 2015 and December 2023 either did not respond to CBZ, experienced an incomplete response, or could not tolerate it, and were subsequently prescribed LCM, GBP, or BAC as second-line treatments for TN-related pain. | 49 | Mean: 62.1 (SD: 14.1) | 53.0 | Median: 200 (IQR: 112) | GBP, Oral  BAC, Oral | 14.3 | 3.4 (7.5) | L3M1S3 |

BAC, baclofen; CBZ, carbamazepine; GBP, gabapentin; ICHD-3, International Classification of Headache Disorders, 3rd edition; IQR, interquartile range; IV, intravenous; LCM, lacosamide; PHT, phenytoin; SD, standard deviation; TN, trigeminal neuralgia; S, C, O, selection, comparability, outcome; L, M, S, low, moderate, and serious risk of bias.

Supplementary Table 3. Sensitivity analysis for safety of lacosamide in patients with trigeminal neuralgia.

| Outcome | Outcome Subgroup | Muñoz-Vendrell *et al*. [20], 2023 and Lappichetpaiboon *et al*. [19], 2024 | | Muñoz-Vendrell *et al*. [9], 2025 and Lappichetpaiboon *et al*. [19], 2024 | |
| --- | --- | --- | --- | --- | --- |
|  |  | *I*² | Proportion (95% CI) | *I*² | Proportion (95% CI) |
| Adverse Events | Sleepiness/Somnolence | 95.8% | 26.7% (0.0%*–*100.0%) | 87.0% | 46.8% (0.0%*–*100.0%) |
|  | Dizziness | 67.7% | 21.4% (1.2%*–*86.2%) | 23.6% | 29.4% (16.6%*–*46.6%) |
|  | Instability | 0.0% | 3.1% (1.0%*–*9.1%) | 0.0% | 0.0% (0.0%*–*100.0%) |
|  | First-Grade AVB | 0.0% | 1.0% (0.1%*–*6.9%) | 0.0% | 2.9% (0.4%*–*18.1%) |
|  | Inattention | 0.0% | 2.0% (0.5%*–*7.8%) | 0.0% | 0.0% (0.0%*–*100.0%) |
|  | Bradycardia | 0.0% | 1.0% (0.1%*–*6.9%) | 0.0% | 0.0% (0.0%*–*100.0%) |
|  | Cutaneous Rash | 0.0% | 1.0% (0.1%*–*6.9%) | 0.0% | 0.0% (0.0%*–*100.0%) |
|  | Diplopia | 0.0% | 1.0% (0.1%*–*6.9%) | 0.0% | 0.0% (0.0%*–*100.0%) |
|  | Insomnia | 0.0% | 1.0% (0.1%*–*6.9%) | 0.0% | 0.0% (0.0%*–*100.0%) |
|  | Itchiness | 0.0% | 1.0% (0.1%*–*6.9%) | 0.0% | 0.0% (0.0%*–*100.0%) |
|  | Nausea | 0.0% | 1.0% (0.1%*–*6.9%) | 0.0% | 0.0% (0.0%*–*100.0%) |
|  | Tremor | 0.0% | 1.0% (0.1%*–*6.9%) | 0.0% | 0.0% (0.0%*–*100.0%) |

AVB, atrioventricular block; CI, confidence interval.

Supplementary Table 4. Certainty of evidence assessment with GRADE framework.

| Certainty assessment | | | | | | | Certainty | Importance |
| --- | --- | --- | --- | --- | --- | --- | --- | --- |
| No. of studies | Study design | Risk of bias | Inconsistency | Indirectness | Imprecision | Other considerations |  |  |
| Pain Relief | | | | | | | | |
| 4 | non-randomised studies | serious^a^ | not serious | serious^b^ | serious^c^ | none | ⨁◯◯◯  Very low^a,b,c^ | CRITICAL |
| Adverse Events (All) | | | | | | | | |
| 3 | non-randomised studies | serious^d^ | serious^e^ | serious^b^ | serious^c^ | none | ⨁◯◯◯  Very low^b,c,d,e^ | IMPORTANT |

a. Two of the studies had unclear or serious risk of bias.

b. Active comparators (*e.g.*, carbamazepine, phenytoin, gabapentin, baclofen) are unique between studies and limits the interpretability of relative effects.

c. Reported outcomes had wide confidence intervals.

d. One of the studies had unclear risk of bias.

e. Large difference in proportion reported between oral and intravenous forms of lacosamide.


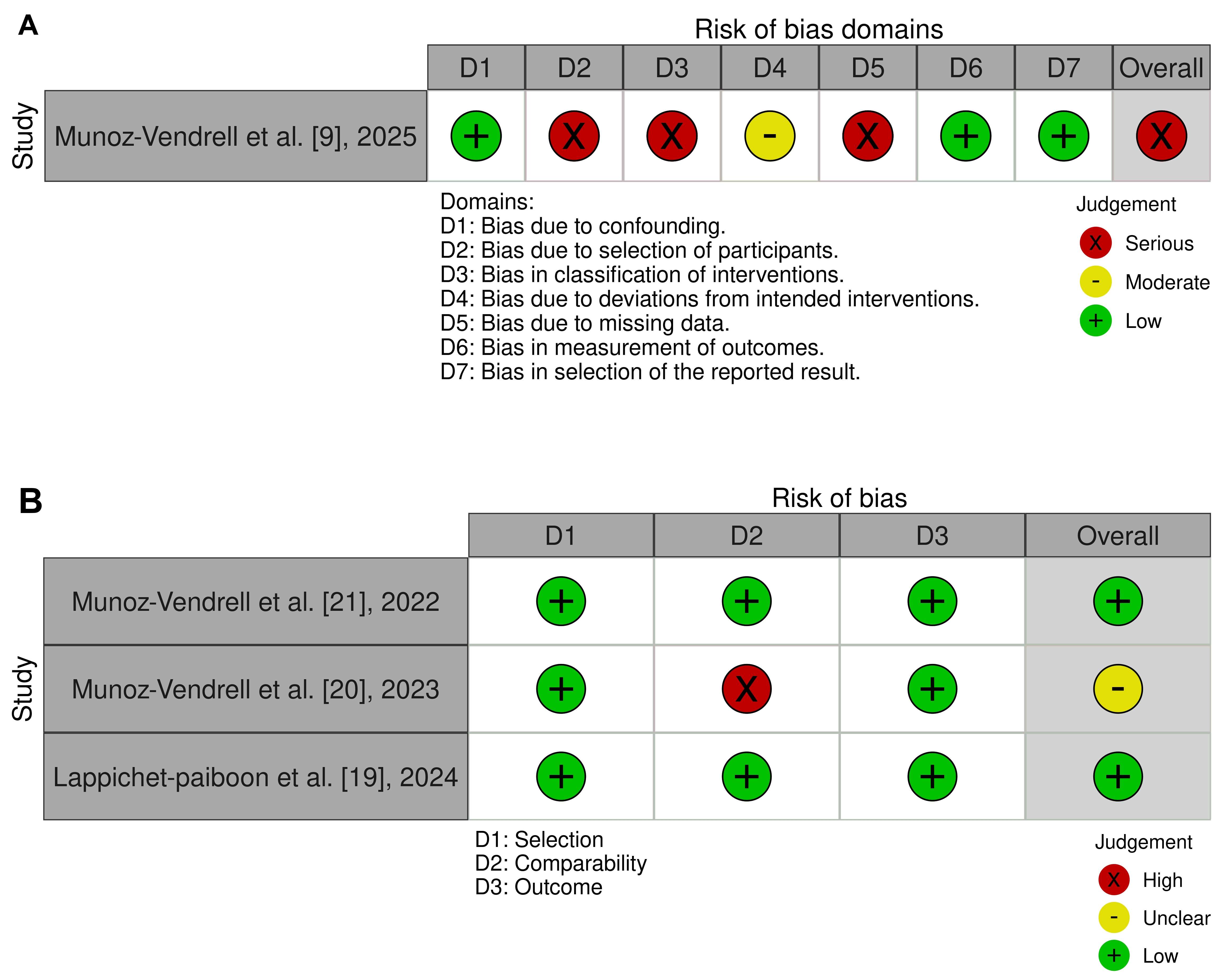


**Supplementary Fig. 1. Traffic light plot for risk of bias assessment.** (A) Risk of Bias in Non-randomized Studies-of Interventions, Version 2. (B) Newcastle-Ottawa Scale for Cohort Studies.


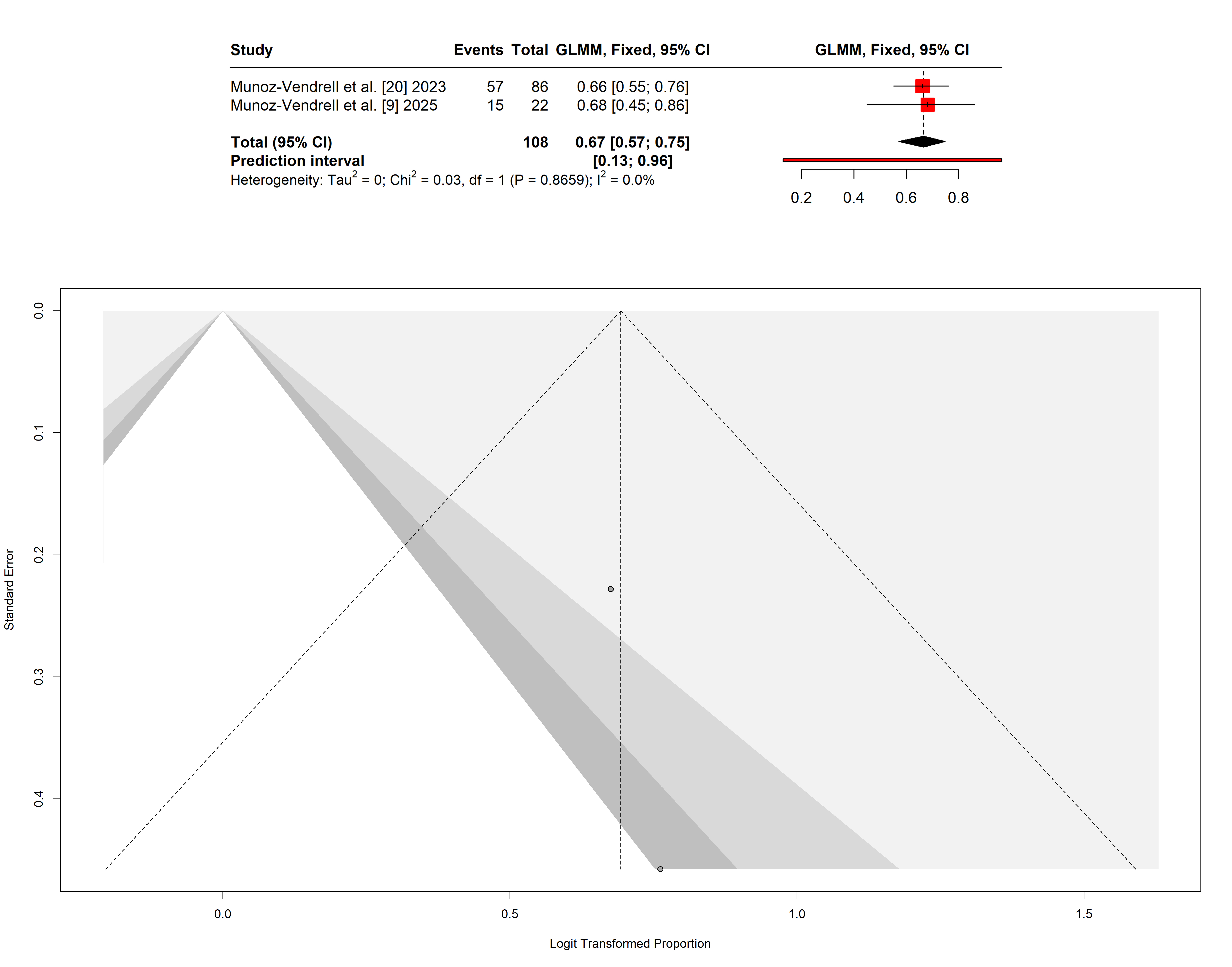


**Supplementary Fig. 2.** **Forest and funnel plots showing the pain relief efficacy of lacosamide in patients with trigeminal neuralgia.** CI, confidence interval; GLMM, Generalized linear mixed models.


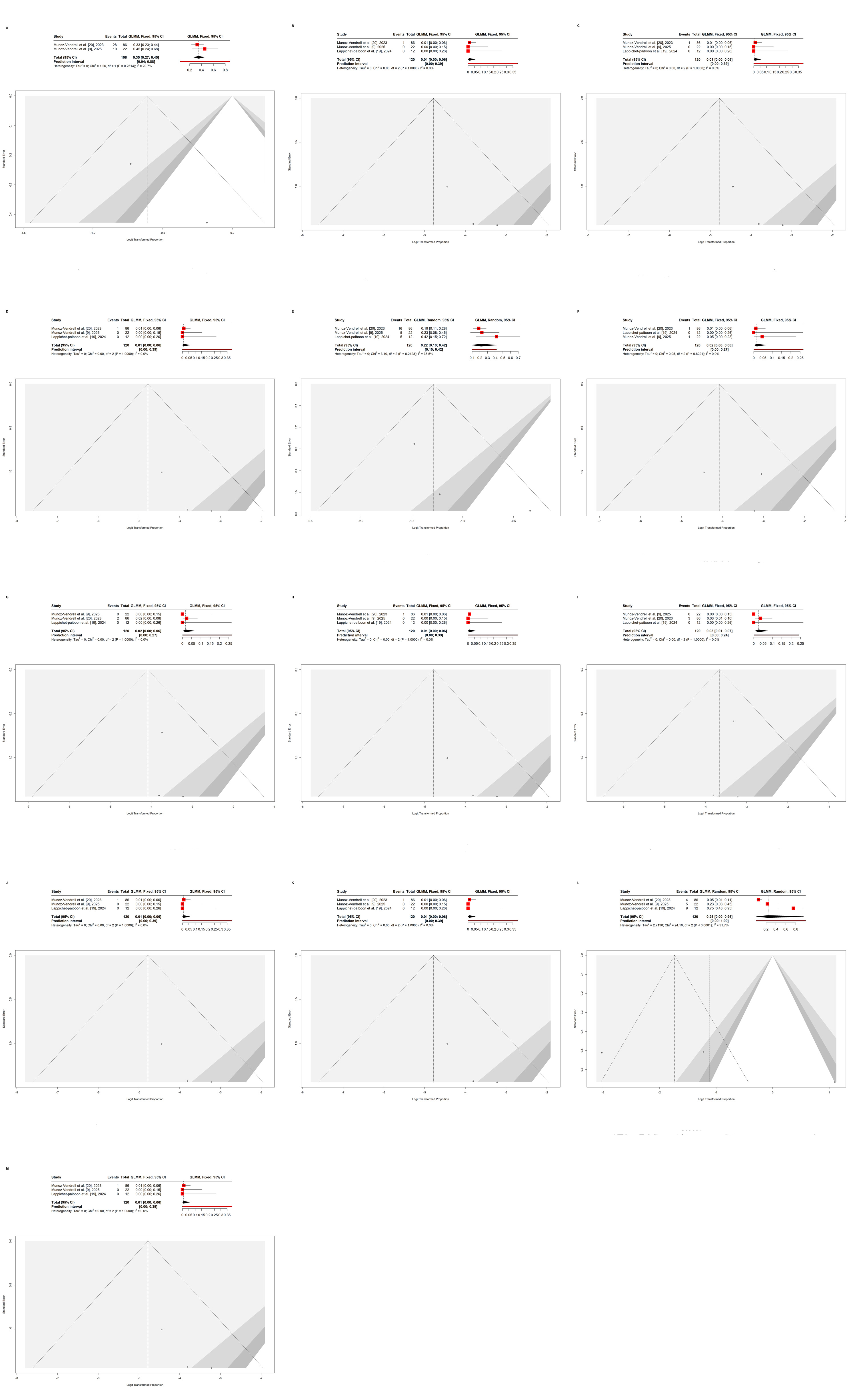


**Supplementary Fig. 3. Forest and funnel plots showing the safety endpoints of Lacosamide in patients with trigeminal neuralgia.** (A) Overall AEs. (B) Bradycardia. (C) Cutaneous rash. (D) Diplopia. (E) Dizziness. (F) First-degree AVB. (G) Inattention. (H) Insomnia. (I) Instability. (J) Itchiness. (K) Nausea. (L) Sleepiness/Somnolence. (M) Tremor. AVB, atrioventricular block; CI, confidence interval; GLMM, Generalized linear mixed models.
